# Agranulocytosis Secondary to Cancer Chemotherapy Associated With Higher In-Hospital Mortality in Patients With Central Line Insertion During a Hospital Stay

**DOI:** 10.7759/cureus.34717

**Published:** 2023-02-07

**Authors:** Mohamed Z Mohamed Jiffry, Aimal Khan, Felipe Carmona Pires, Nkechi A Okam, Jonathan Vargas, Kayvon Moin, Meagan Josephs

**Affiliations:** 1 Internal Medicine, Danbury Hospital, Danbury, USA; 2 Internal medicine, Danbury Hospital, Fairfield, USA; 3 Medicine, American University of the Caribbean, Cupecoy, SXM; 4 Medicine, American University of the Carribbean, Cupecoy, SXM

**Keywords:** neutropenia, central line associated bloodstream infection, in-hospital mortality, central venous catheter, agranulocytosis secondary to cancer chemotherapy

## Abstract

Background

Agranulocytosis secondary to cancer chemotherapy (ASCC) remains a leading cause of morbidity and mortality. Central line-associated bloodstream infections (CLABSI) are also particularly prevalent in these populations and may portend a poorer outcome. Our study serves to investigate the relationship between patients with agranulocytosis secondary to cancer chemotherapy and the insertion of a central venous catheter (CVC) with respect to in-hospital mortality.

Methods and results

We utilized the National Inpatient Survey 2019 database. We utilized the International Classification of Diseases (ICD)-10 CM codes to identify ASCC and other medical comorbidities. We utilized ICD-10 PCS codes to identify CVC insertions. Multivariate logistic regression was utilized to study the effect of CVC insertion on in-hospital mortality. In patients with ASCC, CVC insertion was associated with a higher in-hospital mortality (unadjusted: 11.9% vs. 1%, p<0.001, adjusted OR 19.27, 95% CI 5.84 - 65.6, p<0.001) adjusted for baseline characteristics and other comorbidities. Patients in the study cohort who were older than 70 years of age also had a higher in-hospital mortality relative to younger age groups (adjusted OR 2.31, 95% CI 1.04-5.13, p<0.039).

Conclusion

In patients with ASCC, CVC insertion during hospitalization is associated with higher in-hospital mortality.

## Introduction

Febrile neutropenia is variably defined as a sustained temperature over 38°C with absolute neutrophil counts (ANCs) less than 1000-1500 cells/microliter and is the most feared complication of agranulocytosis [1]. In general, neutropenic fevers develop in approximately 5 to 10% of patients receiving cytotoxic chemotherapy, compared to 20 to 25% of non-leukemic hematologic malignancy patients and 85 to 95% of acute leukemia patients [2]. The development of neutropenic fever is predicated on several patient, disease, and treatment-related factors, and several other well-defined risk factors have been studied regarding the risk for serious complications in patients who have developed febrile neutropenia as well, including validated scoring systems such as the Multinational Association for Supportive Care in Cancer (MASCC) risk index [3].

Factors that have been studied that portend higher risk for the development of febrile neutropenia include but are not limited to age (65 or older), female sex, poor performance status based on medical comorbidities, as well as various disease-related and treatment-related predictors, such as myelophthisis, and failure to administer prophylactic granulocyte-macrophage colony-stimulating factor (GM-CSF) [4-8].

Central-line associated bloodstream infections (CLABSIs) are an important cause of morbidity and mortality worldwide and are estimated to be responsible for up to 90% of these infections [9]. Several host and catheter-related factors play a role in increasing the risk for the development of CLABSI, such as medical comorbidities, extreme age, catheter site care, type, and location of the catheter inserted, to name a few. Neutropenic patients have also been shown to be at high risk, especially with an ANC count of fewer than 100 cells/mm3 [1].

This study aims to better delineate the relationship between neutropenia following chemotherapy and the insertion of a central venous catheter (CVC) with regards to the risk for increased mortality. We utilized the national inpatient sample database, which is the largest publicly available all-payer database in the United States, containing data on more than seven million hospital stays annually.

## Materials and methods

The National Inpatient Sample (NIS) 2019 database was utilized to conduct this study. Data was extracted by utilizing the International Classification of Diseases, 10th Revision (ICD-10) codes. The inclusion criteria for this study were patients with a primary diagnosis of agranulocytosis secondary to cancer chemotherapy (ASCC) who underwent CVC insertion during the hospitalization. Exclusion criteria for this study were patients with iatrogenic pneumothorax secondary to central line insertion, patients with blood product administration, patients with platelet product administration, and patients with sepsis. The primary outcome studied was in-hospital mortality and was identified by the variable 'DIED' from the NIS database.

Data processing and statistical analysis was done using the SPSS version 26 software package (IBM Inc.m Armonk, New York). Categorical data was compared using Pearson's Chi-squared test, and continuous variables were compared using the independent samples t-test. Multivariate regression utilizing binomial logistic regression was performed with in-hospital mortality as the primary outcome. Multivariate regression was conducted to study the effects of CVC insertion on in-hospital mortality, with covariates including age, sex, race, primary payer status, and select medical comorbidities from the Charlson comorbidity index. A p-value of <0.05 was considered significant for all statistical comparisons.

## Results

**Figure 1:**
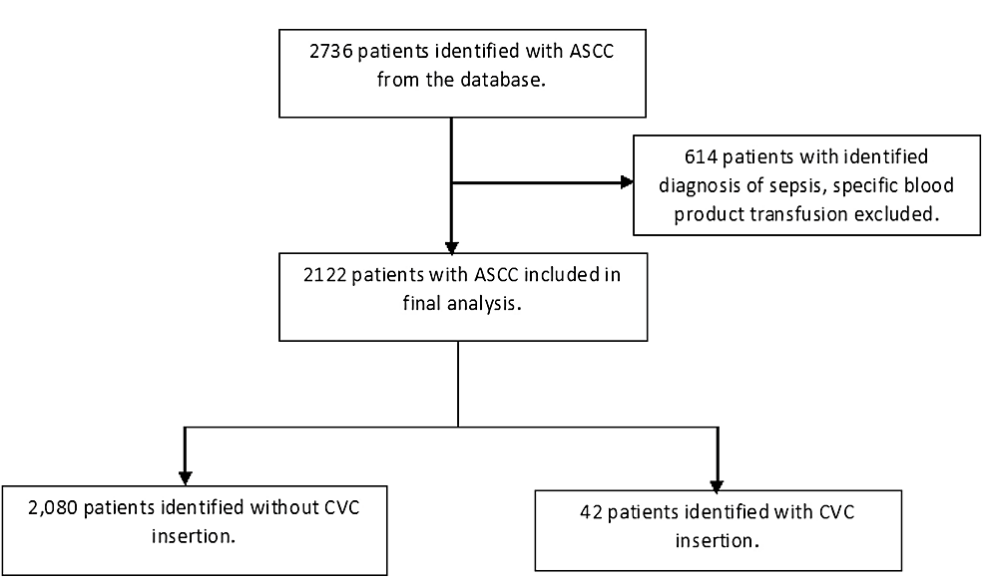
Flowchart demonstrating the study cohort ASCC - agranulocytosis secondary to cancer chemotherapy, CVC - central venous catheter

Two thousand seven hundred thirty-six patients were identified from the database with ASCC. Six hundred fourteen patients who met exclusion criteria were excluded from the study. Two thousand one hundred twenty-two patients with a primary diagnosis of ASCC were included in the analysis. Of these, 2,080 patients were identified who did not undergo CVC insertion, and 42 patients were identified who did (Figure 1).

The baseline characteristics of the cohort of patients identified are listed in Table 1. Unadjusted for other factors, ASCC patients who underwent CVC insertion had a higher in-hospital mortality compared to those who did not undergo CVC placement (11.9% vs. 1%). They also had a higher comorbid incidence of acute kidney injury during the hospitalization (19% vs. 9.4%), and a higher percentage had a history of heart failure prior to hospitalization (14.3% vs. 5.1%). 

**Table 1:** Baseline characteristics of patients with agranulocytosis secondary to cancer chemotherapy with and without CVC placement Values reflect the number of patient records identified within the corresponding category. The percentage of the total number of records studied per category is included within brackets. IQR - inter-quartile range, CVC - central venous catheter

Variable	Without CVC placement (n=2080)	With CVC placement (n=42)
Death in hospital (%)	21 (1)	5 (11.9)
Age, median (IQR)	61 (38-71)	62 (46-70)
Sex (%)		
Male	951 (45.7)	22 (52.4)
Female	1129 (54.3)	20 (47.6)
Race (%)		
White	1462 (70.3)	33 (78.6)
Black	182 (8.8)	6 (14.3)
Hispanic	240 (11.5)	2 (4.8)
Asian/Pacific Islander	59 (2.8)	0 (0)
Native American	11 (0.5)	0 (0)
Other	74 (3.6)	0 (0)
Primary payer (%):		
Medicare	813 (39.1)	18 (42.9)
Medicaid	349 (16.8)	5 (11.9)
Private insurance	822 (39.5)	17 (40.5)
Self-pay	40 (1.9)	1 (2.4)
No charge	2 (0.1)	0 (0)
Other	50 (2.4)	1 (2.4)
Acute kidney injury (%)	196 (9.4)	8 (19.0)
Stroke (%)	3 (0.1)	0 (0)
Ventricular arrhythmia (%)	33 (1.6)	0 (0)
Acute myocardial infarction (%)	10 (0.5)	0 (0)
Diabetes (%)	323 (15.5)	6 (14.3)
Hypertension (%)	685 (32.9)	13 (31)
Chronic kidney disease (%)	186 (8.9)	2 (4.8)
Cirrhosis (%)	17 (0.8)	0 (0)
Old myocardial infarction (%)	61 (2.9)	1 (2.4)
Hx of heart failure (%)	106 (5.1)	6 (14.3)
Hx of peripheral vascular disease (%)	25 (1.2)	0 (0)
Hx of chronic obstructive pulmonary disease (%)	174 (8.4)	1 (2.4)
Hx of dementia (%)	35 (1.7)	0 (0)

In patients with ASCC, patients who had a CVC insertion had a higher in-hospital mortality adjusted for age, sex, race, primary payer status, acute conditions (including acute kidney injury), and pre-existing comorbidities (including history of heart failure) (adjusted OR 19.27, 95% CI 5.84 - 65.6, p<0.001). Adjusted for other covariates, no other comorbidity covariates were significantly associated with increased in-hospital mortality (Table 2).

**Table 2:** Odd's ratios generated from multivariate logistic regression showing the effects of different medical comorbidities and central venous catheter (CVC) insertion on in-hospital mortality in patients with agranulocytosis secondary to cancer chemotherapy CVC - central venous catheter, AKI - acute kidney injury, COPD - chronic obstructive pulmonary disease

Variable	Odds ratio	95% CI lower	95% CI upper	p-value
CVC insertion	19.27	5.84	63.59	<0.001
AKI	2.14	0.81	5.65	0.05
Diabetes	0.94	0.35	2.55	0.606
Hypertension	2.56	0.91	7.16	0.041
Chronic kidney disease	3.08	0.86	10.98	0.038
Hx of heart failure	0.72	0.13	4.11	0.39
Hx of peripheral vascular disease	4.88	0.57	42.04	0.134
Hx of COPD	0.27	0.03	2.15	0.308
Hx of dementia	2.52	0.29	21.56	0.284

Among different age groups (age 30-49, 50-69, and age 70 and above), CVC insertion was particularly associated with increased in-hospital mortality adjusting for other covariates in the age group >70 (adjusted OR 2.31, 95% CI 1.04-5.13, p<0.039). Adjusted for other factors, no significant increase in in-hospital mortality in patients with CVC insertion among the other age groups was noted (Table 3).

**Table 3:** Odds ratios generated from multivariate regression analysis showing the effects of CVC insertion on in-hospital mortality in patients with agranulocytosis secondary to cancer chemotherapy in different age groups CVC - central venous catheter

Age group	Odds ratio	95% CI lower	95% CI upper	p-value
Age >70	2.31	1.04	5.13	0.039
Age 50 - 69	1.18	0.54	2.57	0.687
Age 30 - 49	0.885	0.26	3	0.844

In the study group, a statistically significant difference in the time to undergo the procedure was noted between patients who died during hospitalization and patients who did not (median eight vs. three days, IQR 17.5-3 vs. 5-2 days, mean difference: 6.0 days, 95% CI 9.4 - 2.7 days, p-value<0.001).

In the study group, no statistically significant difference was noted in the total number of procedures undergone during hospitalization and in-hospital mortality (mean difference 2.83, 95% CI -4.3 - 9.97, p-value 0.43). In contrast, a statistically significant difference in the total number of procedures undergone during the hospitalization and in-hospital mortality was noted in the control group (mean difference 0.76, 95% CI 0.38 - 1.14, p-value<0.0001).

## Discussion

Chemotherapy-induced neutropenia remains a leading cause of ICU admission and mortality in patients with both liquid and solid malignancies [10]. Among other identified risk factors, CVC insertion is particularly problematic in this patient population and has been identified as an independent risk factor for the development of CLABSI [11].

CVC insertion is frequently required in the hospital setting for patients with advanced hematologic or solid malignancies, as they can provide reliable access for the administration of chemotherapeutic agents, antibiotics, parenteral nutrition, and blood products. Unfortunately, they can result in serious bloodstream infections, and neutropenia has been associated as an independent risk factor for the development of CLABSIs [8]. Moreover, the crude mortality rate in this patient population is also higher, and the most prevalent potential risk factor was found to be the presence of a central venous line, followed by the presence of peripheral intravenous lines [12]. 

Our study results also suggest that central line insertion in hospitalized patients with ASCC is an independent predictor of increased mortality during the hospital stay. This finding may disproportionately affect younger age groups <70 years of age, as our study also suggests that age >70 is also an independent predictor of increased mortality. This is in keeping with existing data showing that increasing age correlates with a 2.3-fold increase in mortality in critically ill hospitalized patients [13]. Additionally, the fact that there was no significant difference in mortality in the study group regardless of the number of procedures undergone during the hospitalization makes it less likely that the results can be explained by another procedural intervention. However, other procedural interventions may also influence the outcome, as evidenced by a statistically significant difference in outcomes noted in the control group. 

This study has some considerable limitations that need to be accounted for. As an administrative database was utilized, coding errors or omissions may be present, and important patient information such as medication use, laboratory results, provider-dependent factors, and performance status are not available. Other factors that directly influence the outcome, such as the proximate cause of death, the effect of drug interactions, and non-procedural interventions performed, are not accounted for by this database. More importantly, confounding variables that are inadequately captured by this database aside from the comorbidity measures and factors accounted for in this analysis may have disproportionately affected the outcome, such as the initial indication for CVC insertion, patient frailty, type and stage of malignancy, prior treatments received, and withdrawal of care during hospitalization due to patient preference.

It is of some interest to note that the association between CVC insertion and in-hospital mortality persisted despite the exclusion of patients with sepsis in this cohort. Not accounting for the limitations already discussed, the signs and symptoms of infection may be significantly muted in neutropenic patients, and the inability to accurately diagnose infection in this population is also a major cause of morbidity and mortality [14]. 

## Conclusions

ASCC is a potentially life-threatening condition necessitating prompt admission and treatment, with CVC insertion in this cohort being particularly problematic in view of the increased incidence of CLABSI in this population. Our study identified an increased in-hospital mortality in patients who undergo CVC insertion in patients with ASCC, as well as higher in-hospital mortality in patients >70 years of age in this cohort. Clinicians need to remain vigilant about the risks of CVC insertion in patients with ASCC and must maintain a low index of suspicion for CLABSI in view of atypical presentations in this cohort.
